# Engineered P450 biocatalysts show improved activity and regio-promiscuity in aromatic nitration

**DOI:** 10.1038/s41598-017-00897-z

**Published:** 2017-04-12

**Authors:** Ran Zuo, Yi Zhang, Chao Jiang, John C. Hackett, Rosemary Loria, Steven D. Bruner, Yousong Ding

**Affiliations:** 1 Department of Medicinal Chemistry, Center for Natural Products, Drug Discovery and Development, College of Pharmacy, University of Florida, Gainesville, Florida 32610 USA; 2 Department of Pharmaceutical Engineering, School of Chemical Engineering, Nanjing University of Science and Technology, Nanjing, Jiangsu 210094 China; 3 Department of Physiology and Biophysics and the Massey Cancer Center, Virginia Commonwealth University School of Medicine, Richmond, Virginia 23298 USA; 4 Department of Plant Pathology, Institute of Food and Agricultural Sciences, University of Florida, Gainesville, Florida 32611 USA; 5 Department of Chemistry, University of Florida, Gainesville, Florida 32611 USA

## Abstract

Nitroaromatics are among the most important and commonly used chemicals but their production often suffers from multiple unsolved challenges. We have previously described the development of biocatalytic nitration processes driven by an engineered P450 TxtE fusion construct. Herein we report the creation of improved nitration biocatalysts through constructing and characterizing fusion proteins of TxtE with the reductase domain of CYP102A1 (P450BM3, BM3R). The majority of constructs contained variable linker length while one was rationally designed for optimizing protein-protein interactions. Detailed biochemical characterization identified multiple active chimeras that showed improved nitration activity, increased coupling efficiency and higher total turnover numbers compared with TxtE. Substrate promiscuity of the most active chimera was further assessed with a substrate library. Finally, a biocatalytic nitration process was developed to nitrate 4-Me-dl-Trp. The production of both 4-Me-5-NO_2_-l-Trp and 4-Me-7-NO_2_-l-Trp uncovered remarkable regio-promiscuity of nitration biocatalysts.

## Introduction

The nitro (-NO_2_) group acts as an essential unit in a number of pharmaceuticals^1^, exemplified by anticancer drug nilutamine, antiparkinson agent tolcapone, and anti-infective agents chloramphenicol and the recently approved delamanid^2^ and nifurtimox-eflornithine combination^3^. Drug candidates bearing the -NO_2_ group also commonly appear in drug pipelines for treating a variety of existing and emerging diseases^4–6^. Additionally, the nitro group in particular is a versatile synthetic handle present in numerous building blocks in the synthesis of complex drug molecules^7–9^. The fundamental importance of the nitro group in pharmaceutical industry has driven the development of chemical nitration methods^10^. Indeed, syntheses of nitrochemicals, particularly nitroaromatics, are one of the most studied organic reactions^11^, and classical electrophilic nitration methods with nitric acid as the nitrating reagent dominate current industrial processes. The limitations of the electrophilic method, however, is that it is notoriously non-selective, poorly tolerates other functional groups, poses safety concerns, and generates large quantities of acidic waste. Advanced nitration methods^7, 10, 12–15^ have recently been developed to account for these issues but none of them are successful on the scales required for pharmaceutical production.

Developing efficient catalysts that can sustainably produce a broad range of chemicals represents a major challenge in modern organic chemistry. Enzymes as biocatalysts are of great synthetic interest because their typically high stereo-, regio- and chemo-selectivity avoids lengthy protection/deprotection steps and the generation of impurities. Furthermore, biocatalytic processes are often non-toxic, require generally mild reaction conditions, and leave no residual heavy metal contamination^16–18^. As a result, the footprint of enzymes in the industrial production of chemicals is ever-increasing, exemplified by biocatalytic manufacturing of anti-diabetic drug sitagliptin^19^. In these applications, enzymes are capable of catalysing a breadth of organic transformations^20^. However, despite Nature’s successful application of several strategies to synthesize nitro-containing compounds^21^, there has been comparably little effort to exploit them in chemical industry^22^.

We have attempted to expand the biocatalytic toolbox by developing nitration biocatalysts. One example is TxtE, a cytochrome P450 enzyme that uses nitric oxide (NO) and O_2_ to nitrate C4 of the l-tryptophan (Trp) indole in the thaxtomin biosynthetic pathway^23^. It is the only known enzyme enabling direct nitration of a C-H bond in nature, rending it to be a promising lead for developing nitration biocatalysts. P450s catalyse a wide array of oxygenation reactions under mild conditions and have impressive biotechnological potential^17, 24^. However, the requirement of auxiliary redox proteins and low activity and electron coupling efficiency are common limitations that constrain frequent industrial implementation of P450s^25, 26^. Inspired by previous research on artificial self-sufficient systems^27–30^, we have recently addressed the need of redox partners in TxtE applications^22^ by fusing it with the reductase domain (BM3R) of naturally self-sufficient P450 P450BM3^31^. The chimera outperformed TxtE supplemented with spinach ferredoxin (Fer) and ferredoxin reductase (Frd) in terms of catalytic activity, and was subsequently utilized in the biocatalytic syntheses of two fluorinated nitro-Trp analogues^22^. However, both electron coupling efficiency and total turnover number (TTN) of the developed chimeric enzyme were 20% lower than wild type TxtE^22^. These must be addressed to attain our goal of developing efficient TxtE-based nitration biocatalysts for biotechnological applications.

**Figure 1:**
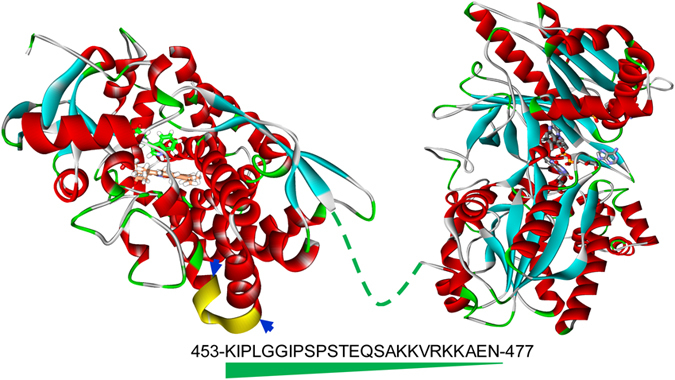
Schematic depiction of chimeric TxtE-BM3R constructs with variable linker length or a swapped loop (yellow). The structure of human NADPH–cytochrome P450 reductase (PDB: 3QE2, right) represented not-available BM3R structure along with TxtE (PDB: 4TPO, left). The 25-AA linker of P450BM3 is shown as a green dash line (middle) along with the amino acid sequence.

Here we describe the characterization of 15 new chimeric TxtE-BM3R biocatalysts. These chimeras were developed by varying the length of a linker connecting TxtE (from *Streptomyces scabies*) and BM3R and swapping a putative interfacial loop on the TxtE to improve interactions with the reductase domain (Fig. 1). These studies have yielded TxtE-BM3R constructs with improved catalytic turnover, coupling efficiency, and broad substrate specificity. These advancements constitute essential steps on the path toward developing advanced nitration biocatalysts for industrial implementation.

## Results and Discussions

### Design and production of chimeric TxtE-BM3R variants

We previously reported three self-sufficient TxtE constructs^22^. Of the two reductase domains that we evaluated, the di-flavin reductase BM3R homologous to eukaryotic cytochrome P450 reductase^32^ conferred superior TxtE nitration activity compared with the P450RhF reductase domain (RhFRED), a natural fusion of Frd and Fer^33^. In the latter case, the catalytic performance was notably dependent on the length of the linker connecting TxtE and RhFRED^22^. Indeed, profound impact of linker length on the performance of artificial fusions has appeared in several recent studies^34–37^. Inspired by these results, we sought to create serial chimeric TxtE-BM3R variants by varying linker length in this work. Using a stepwise cloning approach, chimeric TxtE-BM3R variants with linker lengths of 3, 6, 9, 11, 14, 17, 19, 22, 24, and 27 amino acids (AA) were first constructed (see Supplementary Fig. S1) to quickly assess potential influences of linker length on enzyme nitration performance. To investigate the optimal length, a second set of variants with linker lengths of 12, 13, 15, and 16 was latter prepared by the same cloning method. Hereafter, these TxtE-BM3R variants are designated as TB-linker length, so for example the TxtE-BM3R variant connected by a 3-AA linker is denoted TB3. In all variants, two amino acids glutamate (E) and leucine (L) were appended to *N*-termini of the linkers as a result of the *Sac*I digestion site facilitating molecular cloning. For instance, the linker of TB27 comprised EL and the entire 25-AA linker of P450BM3 (Fig. 1). The previous TxtE-BM3R construct contains a 13-AA linker that bears EQ at its *N*-terminus (Fig. S1)^22^, and is denoted TB13-Q in this study. Superimposition of the crystal structure of TxtE (PDB: 4TPO) with the P450 heme domain of P450BM3 (PDB: 1BVY)^32^ revealed an additional opportunity to potentially improve chimera’s catalytic activity. A loop connecting two helices (J and K, Fig. S2A) of P450BM3 putatively contributes to the interface of heme and reductase domains. This basic-residue-rich loop (A291 to Y313) is about 7 Å away from the acidic residues of the FMN-binding domain and can provide a specific protein-protein interaction for efficient electron transfer (Fig. S2B)^32^. This stretch of amino acids is significantly different from those in TxtE (Fig. S2A). We thereby hypothesized that replacing those residues in TxtE with the corresponding residues in P450BM3 could increase the coupling efficiency between the domains (Fig. 1). The rationally swapped chimera (TB13S) carrying the JK loop from P450BM3 was constructed with overlapping PCR using *TB13-Q* and *P450BM3* as the templates. All constructs were expressed in *E. coli* and purified to homogeneity by a single Ni^2+^-NTA affinity chromatography^22^ (Fig. S3). All chimeras were similarly soluble, indicating the minimal effect of variable linker length on the protein solubility. Except TB3, TB6 and TB9, all chimeras showed a peak at around 450 nm in their reduced-carbon monoxide (CO) difference spectra (Fig. S4)^38^, indicating the production of active P450s. The concentrations of active chimeras and TxtE in purified proteins were quantitated using an extinction coefficient of Δε_450–490nm_ of 91 mM^−1^ cm^−1^ for the ferrous P450–CO complex.

### Nitration performance of chimeric TxtE-BM3R variants

**Figure 2:**
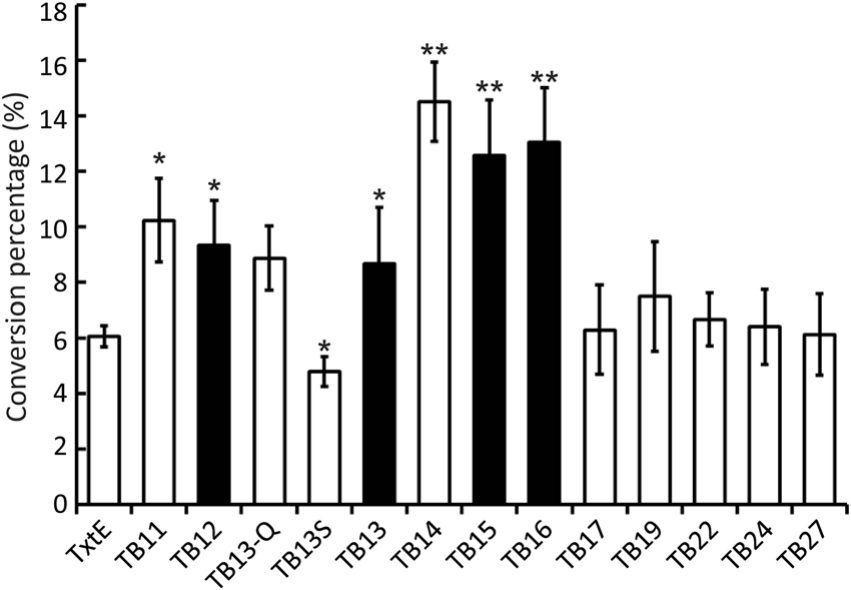
Relative nitration activity of TxtE and chimeric TxtE-BM3R variants. All reactions contained 0.5 mM Trp and 1.5 µM P450. The TxtE reaction was further supplemented with 0.43 µM spinach Fer and 0.33 µM Frd. The reactions were incubated at 20 °C, 300 rpm for 30 minutes. All experiments were repeated at least three times. The results of TB12, TB13, TB15, and TB16 were shown as black bars. Chimeras showed significant difference in nitration activity compared to the wild type TxtE were indicated as *P < 0.05 or **P < 0.01.

We first measured the catalytic performance of TB11, TB13-Q, TB13S, TB14, TB17, TB19, TB22, TB24, and TB27 to quickly assess the potential effects of linker length (Fig. 2). TxtE coupled with spinach Fer and Frd served as the control. HPLC analysis revealed that all fusion enzymes nitrated Trp to a different extent (Figs S5 and 2). After 30 min, TxtE (1.5 µM) converted 6.1% of Trp (0.5 mM) into 4-NO_2_-l-Trp, and this rate was lower than all tested chimeric constructs (1.5 µM). Compared with TxtE, the nitration activity of the most active chimera, TB14, was improved by 2.4 times to reach 14.5% conversion in 30 min. The same was true when the reaction time was extended to 60 min (12.5% for TxtE vs. 28.8% for TB14). Remarkably, TB14 converted 82% of the substrate into the nitro-product after 4 h and its reaction reached the completion at 8 h (Fig. S6). On contrast, the maximal substrate conversion in the TxtE reaction was about 78%, which was achieved after 8 h. The second most active enzyme was TB11 (10.2%), followed by TB13-Q (8.9%). Compared with TxtE, TB13-Q showed a higher initial reaction rate but a lower overall conversion rate (Fig. S6). The nitration activity of chimeras with longer linker length from 17 to 27 AAs were similar (6.2 to 7.5%) and around 2 times lower than TB14 (Fig. 2). Next, we characterized TB12, TB13, TB15, and TB16 to finely examine the optimal length from 11 to 17 AAs (Fig. S1B). The activity of both TB15 and TB16 was higher than TB11 and TB17 and was only slightly lower than TB14 (Fig. 2). On the other hand, TB12 and TB13 retained about 60% of TB14’s activity. These results demonstrated the optimal linker length to be 14 to 16 AAs. Moreover, the similar activity of TB13 and TB13-Q (Fig. S1B) suggested that linker content played a minor role in determining activity (Fig. 2), consistent with the conclusions of several previous reports^35–37^. Additionally, standalone BM3R (Fig. S3) in solution was unable to support TxtE for nitration, indicating the necessity of the linker in modulating proper interactions between TxtE and BM3R.

The nitration activity of rationally designed TB13S (4.8%) was lower than TxtE and TB13-Q (Fig. 2). The observed catalytic activity, however, for the first time demonstrated that TxtE is a robust scaffold for chimerogenesis engineering that tolerates foreign structural elements and has potential to greatly expand fitness and improve catalytic properties of nitration biocatalysts^39, 40^.

To yield mechanistic insights to account for the observed activity changes, we first assessed the binding affinities of Trp toward all active chimeras by UV-Vis difference spectroscopy (Table 1). All fusions showed Type I spectral shift of the Soret peak from 420 to around 390 nm after adding Trp, and demonstrated the overly similar level of binding affinity (K_d_ = ~20 μM). This result indicated that linker length or structural swapping minimally influenced substrate binding. We next determined coupling efficiency to evaluate the extent to which linker length and structural swapping may affect electron transfer compatibility during nitration reaction (Table 1). Coupling efficiency of TB11 (5.2%) and TB14 (5.3%) were about 2.2 times higher than TxtE (2.4%), possibly indicating that improvement in their activity was driven by more effective electron transfer. TB15 (3.9%) and TB16 (2.7%) also showed improved coupling efficiency in comparison with TxtE. Coupling efficiency of the other fusions was similar to that of TxtE. Of note, the positive influence of the swapped JK loop from P450BM3 was reflected by 1.3-time improvement in electron transfer efficiency of TB13S (2.6% vs 2.0% of TB13-Q). Furthermore, the NADPH consumption rate of TB13S was the lowest among all active chimeras and TxtE, and was more than 2 times lower than TB13 and TB13-Q. However, the coupling efficiency of even the most active chimera is comparably low^35, 41^, indicating the great potential of further advancing nitration biocatalysts. Additionally, we determined total turnover number (TTN) as nmol product per nmol P450 in all reactions (Table 1). TB14 had the highest TTN at 707 that was 1.9 times and 2.3 times of values of TxtE and TB13-Q, respectively. TTNs of TB11, TB15, and TB16 were also substantially higher than TxtE, presumably reflecting their higher nitration activity (Fig. 2). We also observed a high TTN of TB27. On the other hand, TTN of TB13S decreased from 308 of TB13-Q to 202, indicating the less efficient use of electrons in its nitration.

**Table 1:** Binding affinity toward Trp, coupling efficiency, NADPH consumption and total turnover numbers of TxtE and its chimeras.

Chimeras	K_d_ (µM)	Coupling (%)	NADPH consumption (µM/min)	TTN
TxtE	24.8 ± 1.1	2.4 ± 0.3	42.3 ± 4.2	378 ± 17
TB11	16.2 ± 0.6	5.2 ± 0.5	32.8 ± 5.0	535 ± 28
TB12	22.3 ± 1.4	1.8 ± 0.2	86.3 ± 3.9	332 ± 40
TB13-Q	21.5 ± 0.8	2.0 ± 0.1	73.9 ± 3.8	308 ± 18
TB13S	19.3 ± 0.6	2.6 ± 0.2	30.8 ± 3.1	202 ± 20
TB13	21.3 ± 0.4	2.2 ± 0.3	65.7 ± 4.1	342 ± 29
TB14	17.4 ± 1.0	5.3 ± 0.5	45.5 ± 4.8	707 ± 16
TB15	21.4 ± 0.9	3.9 ± 0.4	53.6 ± 5.1	658 ± 33
TB16	18.9 ± 1.0	2.7 ± 0.2	80.5 ± 4.3	351 ± 39
TB17	15.5 ± 0.6	2.1 ± 0.3	50.1 ± 4.6	348 ± 24
TB19	16.0 ± 0.8	2.4 ± 0.5	52.1 ± 6.7	464 ± 21
TB22	27.1 ± 1.3	2.4 ± 0.5	46.3 ± 6.3	408 ± 32
TB24	14.9 ± 0.3	2.3 ± 0.1	46.4 ± 3.4	381 ± 15
TB27	16.2 ± 1.1	2.0 ± 0.6	51.1 ± 7.2	548 ± 17

All experiments were performed at least three times.

### Substrate scope of TB14

Next, we attempted to characterize the substrate promiscuity of the most active chimera TB14 as well as TxtE with a library of 29 chemicals that carry substitutions and moderate alterations on the amine, carboxylate, or indole moieties of Trp (Fig. S7). Among these chemicals, 19 has been tested in a recent report with only wild type TxtE^42^. In spectroscopic analysis, 20 Trp analogues (Fig. S7A) induced the Type I spectral shift of TB14 indicating binding, while simple aromatics such as indole and other aromatic amino acids (Fig. S7B) had no detectable level of interaction with the enzymes. There was no significant difference between TB14 and TxtE in terms of binding affinities (Table S1, Figs S8 and S9), further confirming the minimal effect of fused BM3R on substrate binding^22^ (Table 1). Compounds with modified amine or carboxylate moiety generally showed weaker interactions than those with substitutions on the indole ring, the same observed previously^42^. We then examined the nitration activity of TB14 and TxtE toward all library members. To generate sufficient products from less favourable substrates for HPLC detection, the reactions were incubated for 45 min. Besides Trp and α-Me-dl-Trp, both enzymes nitrated seven Trp analogues with substitutions on the C4, C5, C6, and C7 of the indole (Figs S7A and 3). TB14 showed the higher activity toward all substrates. After 45 mins, both enzymes nitrated a significant amount of 4-Me-dl-Trp, 5-Me-dl-Trp, 5-F-l-Trp and 6-F-dl-Trp but not α-Me-dl-Trp, 4-F-dl-Trp, 5-MeO-dl-Trp and 7-Me-dl-Trp. 5-F-l-Trp emerged as the best substrate of TxtE and was comparable to Trp as the best in the TB14 reaction. Since enzymes nitrate only the l-conformers of racemic substrates, a higher enzyme activity can be expected with enantiopure substrates. The quantitative measurement of both TxtE and TB14 toward the select analogues supported improved performance of TB14 across a variety of substrate analogues and underlined its immediate applications to produce unnatural nitro-Trp compounds. These compounds can have broad applications in synthesizing numerous bioactive peptidic compounds and in analysing macromolecule structures and dynamics^43^.

**Figure 3:**
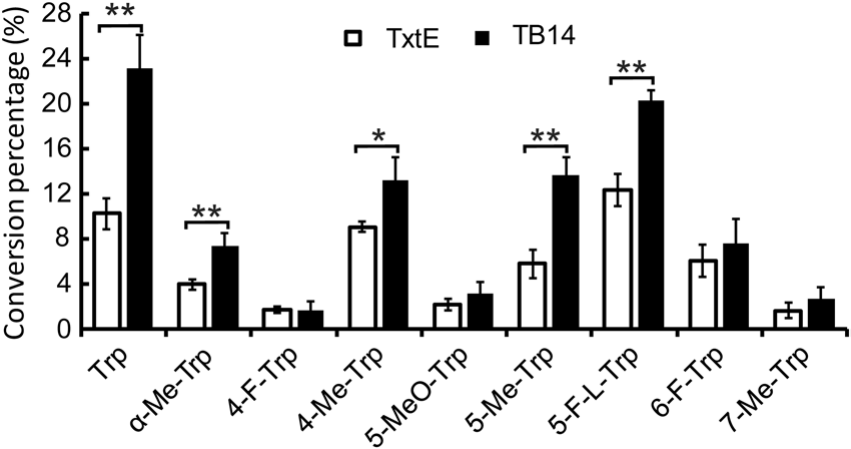
TxtE and TB14 nitrated Trp and its analogues to varying degrees. The reactions were prepared as described previously and incubated at 20 °C, 300 rpm for 45 minutes. All experiments were repeated at least three times. Substrates showed significant difference in the TxtE and TB14 reactions were indicated with *P < 0.05 or **P < 0.01.

### Regio-promiscuity of TB14 in nitration

In previous study, we have observed remarkable, substrate-tuned regio-specificity of TB13-Q that selectively nitrates C7 when C4 of its substrate indole is occupied by an F substitution^22^. To further probe the extent to which physiochemical properties of the substituted group at C4 impact enzyme regio-selectivity, we employed TB14 in a scaled-up reaction to nitrate commercially available racemic 4-Me-dl-Trp. Compared with the fluorine replacement, the methyl group is larger in size and is electron donating in nature. Interestingly, the binding affinity of 4-Me-dl-Trp was 13 times higher than 4-F-dl-Trp (Table S1), and TB14 favoured 4-Me-dl-Trp about 8 times more over 4-F-dl-Trp in nitration (Fig. 3).

Despite technical challenges associated with production purification, we isolated about 80 µg of nitrated 4-Me-dl-Trp as a yellow powder by semi-preparative HPLC. By Marfey’s derivatization, we observed the significant consumption (~85%) of 4-Me-l-Trp in the reaction and identified the nitro product with l-configuration (Fig. S10). This result further confirmed the strict stereo-selectivity of TB14 in nitration. LC-high resolution (HR) MS analysis of the isolated product confirmed the nitration on the substrate by giving one major peak with the expected molecular weight of nitro product (m/z = 264.0892, Fig. S11). Interestingly, one minor peak with the same molecular weight was eluted right after the major one (Fig. S11), suggesting the coexistence of two structural isomers in the product sample.

**Figure 4:**
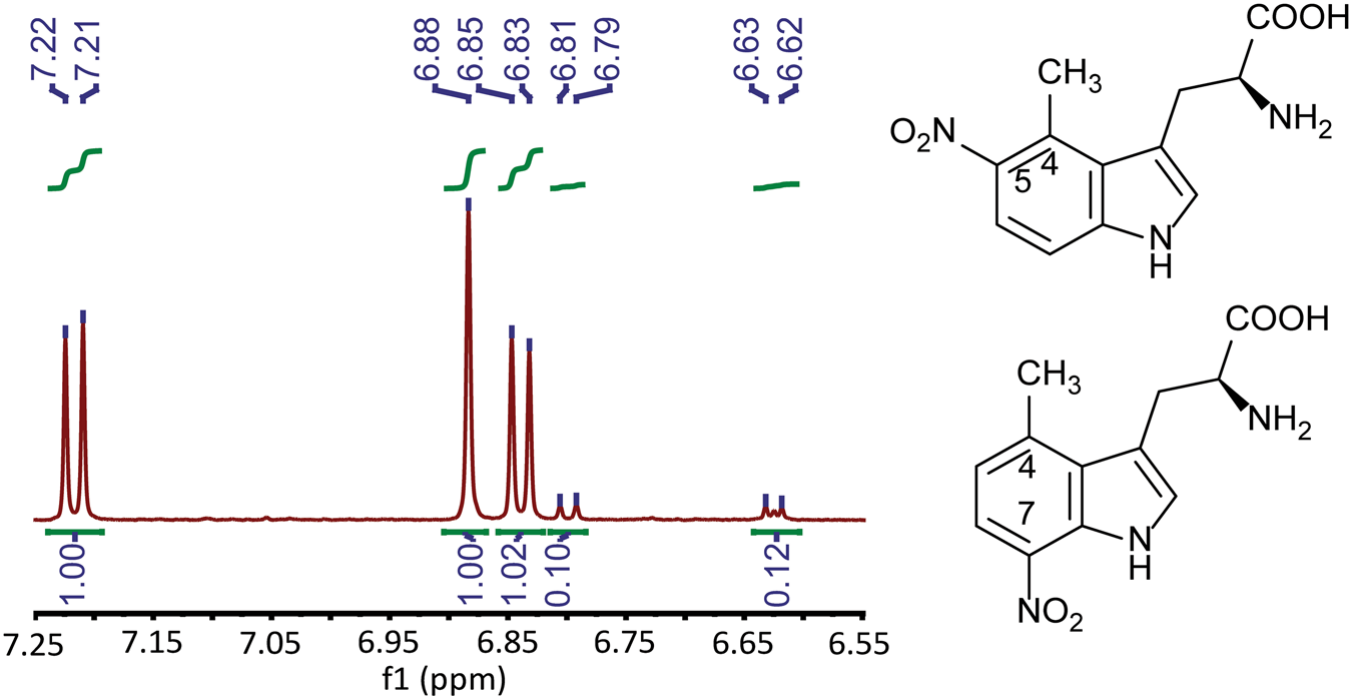
Both 4-Me-5-NO_2_-l-Trp and 4-Me-7-NO_2_-l-Trp were produced in the TB14 reaction with 4-Me-dl-Trp as substrate. The aromatic region of ^1^H NMR spectrum of isolated product showed the chemical shifts of two sets of aromatic protons. Integrated values of these protons were also included.

Next, we structurally characterized the isolated product by ^1^H and ^13^C and 2D NMR analysis (Figs S12–S17). From the ^1^H and COSY NMR spectra (Figs S12 and S13), we noticed two well separated AX coupling systems in the aromatic region, presumably indicating two nitration sites in the indole. One AX coupling system involved two large doublet peaks at δ 7.22 ppm and 6.84 ppm (Fig. 4) while the other was from another set of two small doublet peaks at δ 6.80 ppm and 6.62 ppm (Fig. 4). The large vicinal coupling constants (8–9 Hz) of the two AX coupling systems suggested the C5 and C7 of the indole as the nitration sites (Table S2, Fig. 4). Two additional AMX coupling systems occurring in the aliphatic region further provided details about the existence of two sets of α and β protons of Trp derivatives (Figs S12 and S13). Given the larger deshielding effect of C5-NO_2_ than C7-NO_2_ on the C4-Me group, the methyl signal of 4-Me-5-NO_2_-l-Trp was in the lower field (δ 2.22 ppm vs 2.10 ppm in 4-Me-7-NO_2_-l-Trp, Table S2). Using the integration values of two methyl groups, we determined the molar ratio of 4-Me-5-NO_2_-l-Trp: 4-Me-7-NO_2_-l-Trp to be about 10:1 (Figs S12 and 4). We were also able to observe the chemical shift signals of 4-Me-5-NO_2_-l-Trp in the ^13^C NMR spectrum (Table S2, Fig. S14). The determination of the product structure was then assisted by HSQC and HMBC spectra (Figs S15 and S16). In the HMBC spectrum, the C4-Me group (2.22 ppm) correlated with a significantly deshielded aromatic carbon (142.18 ppm) that became possibly only by the C5-NO_2_ group (Figs S16 and S17). Therefore, TB14 carried a striking regio-flexibility in nitrating 4-Me-dl-Trp to produce predominantly 4-Me-5-NO_2_-l-Trp and 4-Me-7-NO_2_-l-Trp as a minor product (Fig. 4). These results uncovered that different types of substituted groups at C4 of the indole ring can affect key parameters (activity and regio-selectivity) of TxtE biocatalysts. Interestingly, a single residue His176 in the F/G loop of TxtE was computationally identified as a potential determinant of enzyme regio-selectivity, and engineering of this site to some residues (e.g., Phe and Tyr) indeed created TxtE mutants that predominantly produce 5-NO_2_-l-Trp^44^. Our results provided new insights into intriguing and synthetically important TxtE’s regio-selectivity.

## Conclusions

In summary, we created 15 new chimeric TxtE fusion constructs by employing two protein engineering strategies in an attempt to develop novel biocatalytic nitration processes. Rationally swapping the JK loop of TxtE with that of P450BM3 noticeably improved the coupling efficiency of resulted variant (TB13S) but not its overall nitration performance. Varying the linker length between TxtE and BM3R led to the creation and identification of multiple chimeras, particularly TB14, which showed significantly higher catalytic activity than TxtE and TB13-Q^23^. Remarkably, TB14 is the most active aromatic nitration biocatalyst ever developed. TB14 is also noteworthy in having broad substrate scope, suggesting its promising uses to synthesize a variety of nitro Trp analogues for biomedical and biological applications. Furthermore, we demonstrated the production of both 4-F-5-NO_2_-l-Trp and 4-Me-7-NO_2_-l-Trp by the TB14-driven process. The demonstrated ability of TB14 to nitrate two sites of 4-Me-l-Trp marked TxtE as the promising material for developing nitration biocatalysts to synthesize structurally diverse nitroaromatics via protein engineering. Structural and mechanistic studies to clarify the molecular determinants for TxtE’s substrate scope and regio-specificity are under way and can expand the fundamental understanding of enzyme evolution and mechanism and guide further engineering efforts.

## Materials and Methods

### General Chemicals, DNA Sub-cloning, and Bacterial Strains

Molecular biology reagents and enzymes were purchased from Fisher Scientific. Primers were ordered from Sigma-Aldrich. 4-Me-dl-Tryptophan was from MP Biomedical (Santa Ana, CA), while NOC-5 (3-(Aminopropyl)-1-hydroxy-3-isopropyl-2-oxo-1-triazene) was purchased from EMD Millipore. Other chemicals and solvents were purchased from Sigma-Aldrich and Fisher Scientific. *Escherichia coli* DH5α andBL21-GOLD (DE3) (Agilent) were used for routine molecular biology studies and protein expression, respectively, and were grown in Luria-Bertani broth or Terrific broth. DNA sequencing was performed at Eurofins. A Shimadzu Prominence UHPLC system (Kyoto, Japan) fitted with an Agilent Poroshell 120 EC-C18 column (2.7 µm, 3.0 × 50 mm), coupled with a PDA detector was used for HPLC analysis. For semi-preparative HPLC, YMC-Pack Ph column (5 µm, 4.6 × 250 mm) was used. All NMR spectra were recorded in 100 mM DCl in D_2_O on a Bruker 600 MHz spectrometer using a 5 mm TXI Cryoprobe in the AMRIS facility at the University of Florida, Gainesville, FL, USA. The instrument was operated at 600.17 MHz for ^1^H NMR and 150.9 MHz for ^13^C NMR. Spectroscopy data were collected using Topspin 3.5 software. HRMS data were obtained using a Thermo Fisher Q Exactive Focus mass spectrometer equipped with electrospray probe on Universal Ion Max API source.

### Construction of TxtE-BM3R variants

*TxtE* gene was amplified from genomic DNA of *S. scabies* 87.22 (NRRL B-24449) using a pair of SELKnco-F and SELKsac-R primers in PCR reactions (Table S3). The PCR product was analyzed by agarose gel and extracted with a GeneJET Gel Extraction Kit (Thermo). Purified PCR products and pET28b were digested with the restriction enzymes *Nco*I and *Sac*I, and corresponding linear DNAs were ligated to generate expression construct pET28b-TxtEs. To further create the TxtE-BM3R variants with variable linker length, BM3R domain with selected linker lengths was amplified from *P450BM3* gene by a set of primer pairs (Table S3). Purified PCR products and pET28b-TxtE construct were then digested with the restriction enzymes *Sac*I and *Xho*I, and corresponding linear DNAs were ligated to generate pET28b-TxtE-BM3R expression constructs (Fig. S1). To create the P450BM3 standalone reductase (BM3R) expression constructs, *BM3R* gene was amplified using a pair of BM3R-F and BM3R-R primers. Purified PCR products and pET28b were digested with the restriction enzymes *Nde*I and *Xho*I, and corresponding linear DNAs were ligated to create pET28b-BM3R. To create TB13S fusion variant, we used *TB13-Q* as the template and amplified the TxtES fragment with primers of TxtESF and TxtESR (Table S3), and the S13BM3R fragment with primers of FW-V1 and RV (Table S3) in PCR reactions. P450BM3 gene was used as the template to amplify JKL insert using primers of V1-3-F and V1-3-R. After purification, these fragments were fused by overlapping PCR technology. The full-length *TB13S* was cloned into the expression vector as described above. All inserts in the constructs were sequenced to exclude mutations introduced during PCR amplification and gene manipulation.

### Heterologous expression and purification of recombinant proteins

Protein expression and purification followed our previous protocols^22^. The purified proteins were exchanged into storage buffer (25 mM Tris-HCl, pH8.0, 100 mM NaCl, 3 mM βME, and 10% glycerol) by PD-10 column, aliquoted and stored at −80 °C until needed. CO difference spectroscopy was used to measure the concentrations of functional P450s^38^.

### Spectral analysis of chimeric TxtE-BM3R variants

Purified TxtE and its chimeric fusions were spectrally analyzed following a previous protocol^22^. Briefly, the absorbance spectra (400–600 nm) of TxtE and its chimeric fusions in Tris-HCl (25 mM, pH 8) buffer were recorded by a Shimadzu UV2700 dual beam UV-Vis spectrophotometer. The ferric heme of enzymes was then saturated by bubbling carbon monoxide (Airgas) and the spectra of the saturated enzyme solutions were recorded. Sodium dithionite solution (30 μL, 0.5 M) was then added to reduce ferric ion, and reduced spectra were taken subsequently. CO reduced difference spectra of all P450s were created by subtracting the CO binding spectra from the reduced spectra. Data were further analyzed by Excel. Substrate binding affinities to P450s were measured using 1.5 μM of enzyme solutions in 25 mM Tris-HCl, pH 8.0^22^. The changes in absorbance (ΔA) were determined by subtracting the absorbance at ~420 nm from that at ~390 nm. Data were then fitted to the equation of ΔA = ΔA_max_[L]/(K_d_ + [L]) using GraphPad Prism 4.

### Catalytic activities of chimeric TxtE-BM3R variants

P450 reactions (100 μl) contained 0.5 mM substrate, 1 mM NADP^+^, 1 mM glucose, ~10 units/mL self-prepared glucose dehydrogenase crude extract, 1 mM NOC-5 in 100 μL of Tris-HCl buffer (100 mM, pH 8.0). As the positive control, the TxtE reaction was also re-constructed in the above mixture further supplemented with 0.43 µM spinach Fer and 0.33 µM Frd. The reactions were initiated by adding 1.5 µM P450s, and incubated at 20 °C, 300 rpm on a thermostat (Eppendorf) for 30 or 45 minutes. Methanol (200 μl) was then added to stop the reactions. After centrifugation, 10 μl solutions were analyzed by HPLC. The 4-NO_2_-l-tryptophan was synthesized in a large-scale enzymatic reaction to establish a standard curve for product quantification. To determine the coupling efficiency, NADPH (2 mM) was used to replace the NADPH regeneration system in the reaction mixture. NADPH consumption in enzyme reactions was measured at 340 nm (ε = 6.22 mM^−1^cm^−1^) with a Biotek Synergy HT Multi-Detection Microplate Reader. Non-enzymatic oxidation of NADPH was subtracted as the background. The quantity of nitrated product was determined by HPLC analysis as described above. Coupling efficiency (%) was determined as product (nmol)/consumed NADPH (nmol) × 100%. All reactions were independently repeated at least three times. In TTN and coupling efficiency studies, 0.5 µM P450s and 0.5 mM substrate were used. Total turnover number (TTN) was reported as nmol product per nmol P450. Conversion rate (%) was calculated as product (nmol)/(product + substrate) (nmol) × 100%. All experiments were performed at least in triplication.

### Large-scale enzymatic synthesis of nitrated Trp analogs

To isolate sufficient amount of nitrated 4-Me-Trp analogues for structural determination, 18 μM TB14 was used in a 10-mL reaction mixture containing 1.5 mM 4-Me-Trp, 3 mM NADP^+^, 3 mM glucose, ~30 units/mL self-prepared glucose dehydrogenase crude extract, 3 mM NOC-5 in 100 mM Tris-HCl buffer (pH 8.0). The reactions in a 200-ml flask were incubated at 20 °C, 250 rpm overnight, and then terminated by 20 mL methanol or acidification to pH 1.0 with 6 M HCl. After centrifugation, the supernatants were concentrated in vacuo and then freeze-dried. The products were redissolved in 3 ml methanol for semi-preparation^22^.

### Analytical and semi-preparative HPLC analysis

For analytical analysis, the HPLC column kept at 40 °C. Solvent A and B were water with 0.1% formic acid and acetonitrile with 0.1% formic acid, respectively. The column was eluted first with 1% solvent Bfor 1 min and then with a linear gradient of 1–20% solvent B in 8 min, followed by another linear gradient of 20–99% solvent B in 2 min. The column was further cleaned with 99% solvent B for 2 min and then re-equilibrated with 1% solvent B for 2 min. The flow rate was set as 1 mL/min, and the products were detected at 211 nm with a PDA detector. For semi-preparative analysis, the column was first eluted with 20% solvent B (acetonitrile with 0.1% formic acid) for 3 min and then with a linear gradient of 20–40% solvent B for 3 min, followed by a linear gradient of 40–99% solvent B for 6 min. The column was then cleaned by 99% solvent B for 2 min and re-equilibrated with 20% solvent B for 3 min. The flow rate was set at 3 mL/min, and the products were detected at 211 nm with a PDA detector. All isolates were combined, concentrated, freeze-dried, and then weighed.

### NMR analysis

In NMR analysis, chemical shifts were reported in parts per million (ppm) downfield from tetramethylsilane. Proton coupling patterns were described as singlet (s), doublet (d), double doublet (dd), triplet (t), and multiplet (m). 4-Me-5-nitro-l-tryptophan: ^1^H NMR (600 MHz, 100 mM DCl in D_2_O) δ 7.22 (d, *J* = 9.0 Hz, 1H), 6.88 (s, 1H), 6.84 (d, *J* = 8.9 Hz, 1H), 3.80 (dd, *J* = 10.1, 5.1 Hz, 1H), 3.22 (dd, *J* = 15.6, 5.1 Hz, 1H), 2.84 (dd, *J* = 15.7, 10.1 Hz, 1H), 2.23 (s, 3H). ^13^C NMR (151 MHz, D_2_O) δ 170.56, 154.71, 142.20, 138.27, 128.39, 127.74, 124.17, 118.79, 109.77, 109.69, 58.96, 53.66, 27.77, 15.15. HRMS (ESI+): calc. for C_12_H_13_N_3_O_4_ [M + H]^+^: 264.0906, found: 264.0892. 4-Me-7-nitro-l-tryptophan: ^1^H NMR (600 MHz, 100 mM DCl in D_2_O) δ 6.80 (d, *J* = 8.3 Hz, 1H), 6.63 (d, *J* = 8.1 Hz, 1H), 3.92 (dd, *J* = 10.7, 5.4 Hz, 1H), 3.22 (dd, *J* = 16.2, 5.4 Hz, 1H), 2.98 (dd, *J* = 16.2, 10.8 Hz, 1H), 2.10 (s, 3H). HRMS (ESI+): calc. for C_12_H_13_N_3_O_4_ [M + H]^+^: 264.0906, found: 264.0893.

### Marfey’s derivatization

4-Me-Trp and nitrated 4-Me-Trp from enzyme reactions were reacted with Marfey’s reagent following manufacture manual (Thermo Scientific). Derivatized products were analyzed by LC-MS with A SHIMADZU Prominence UPLC system fitted with a Waters SymmetryShieldTM RP-C18 column (3.5 µm, 4.6 × 100 mm) and a Linear Ion Trap Quadrupole LC/MS/MS Mass Spectrometer system. The flow rate was 0.5 mL/min. The column was eluted with 90% solvent A (0.05 M triethylammonium acetate, pH 3.0), 10% solvent B (acetonitrile) for 2 min and then with a linear gradient of 10–50% solvent B for 60 min. The column was then cleaned by 50% solvent B for 5 min followed by a re-equilibration with 10% solvent B for 2 min. For MS detection, the turbo spray conditions used were: curtain gas: 30 psi; ion spray voltage: 5000 V; temperature: 550 °C; ion source gas 1: 30 psi; ion source gas 2: 20 psi.

### Statistical analysis

Student’s t-test was used to determine if the nitration activity of TxtE and its chimeras are significantly different from one another. To be considered as being significant, P is <0.05.

## Electronic supplementary material

revised supporting information
