# Supplementary material for: Engineered P450 biocatalysts show improved activity and regio-promiscuity in aromatic nitration
Source: Sci Rep. 2017 Apr 12;7:842. doi: 10.1038/s41598-017-00897-z (PMC5429796; doi:10.1038/s41598-017-00897-z)
Supplement: Supplementary file 1 — revised supporting information [file 41598_2017_897_MOESM1_ESM.pdf]

Engineered P450 biocatalysts show improved activity and regio-promiscuity in aromatic nitration

Ran Zuo,[a] Yi Zhang,[a] Chao Jiang,[b] John C. Hackett,[c] Rosemary Loria,[d] Steven D. Bruner[e] and Yousong Ding*[a]

[a]Department of Medicinal Chemistry, Center for Natural Products, Drug Discovery and Development, College of Pharmacy, University of Florida, Gainesville, Florida, 32610, USA

[b]Department of Pharmaceutical Engineering, School of Chemical Engineering, Nanjing University of Science and Technology, Nanjing, Jiangsu 210094, China

[c]Department of Physiology and Biophysics and the Massey Cancer Center, Virginia Commonwealth University School of Medicine, Richmond, Virginia, 23298, USA

[d]Department of Plant Pathology, Institute of Food and Agricultural Sciences, University of Florida, Gainesville, Florida, 32611, USA

[e]Department of Chemistry, University of Florida, Gainesville, Florida, 32611, USA

Corresponding Author:

Yousong Ding

Department of Medicinal Chemistry, Center for Natural Products, Drug Discovery and Development, College of Pharmacy, University of Florida, Gainesville, USA.

E-mail: ydinq@cop.ufl.edu

Table S1: Binding affinities of 20 Trp analogues toward TxtE and TB14[a]

Substrate analogs	TxtE K_d (μM)	TB14 K_d (μM)
L-Trp	25 ± 1	17 ± 1
D-Trp	430 ± 17	470 ± 25
Indole-3-pyruvic acid	390 ± 43	480 ± 32
Indole-3-lactic acid	> 7 mM	> 7 mM
3-Indoleacetic acid	> 17 mM	> 17 mM
Tryptamine	68 ± 6	60 ± 8
Serotonin	320 ± 31	310 ± 39
Indole-3-carboxylic acid	> 9 mM	> 9 mM
2,3,4,9-Tetrahydro-1H-β-carboline-3-carboxynate	580 ± 44	730 ± 86
L-tryptophanol	220 ± 32	280 ± 43
α-Me-Trp	100 ± 4	95± 9
7-Azatryptophan	17 ± 1	26 ± 1
4-F-Trp	190 ± 11	180 ± 13
4-Me-Trp	10 ± 1	14 ± 1
5-OH-L-Trp	340 ± 26	450 ± 16
5-MeO-Trp	29 ± 1	26 ± 2
5-Me-Trp	35 ± 1	30 ± 1
5-F-L-Trp	84 ± 4	78 ± 6
6-F-Trp	73 ± 6	65 ± 5
7-Me-Trp	13 ± 1	11 ± 1

[a]: Binding affinities of chemicals to P450s were measured using 1.5 μM of enzymes in 25 mM Tris-HCl, pH 8.0. All experiments were repeated at least three times. Data were fitted to the Michaelis-Menten equation.

Table S2: ^{13}C and ^{1}H NMR data of 4-Me-5-NO_2-L-Trp and 4-F-7- NO_2- L-Trp and ^{1}H NMR data of 4-Me-7-NO_2-L-Trp

Atom	4-Me-5-NO_2-L-Trp		4-Me-7-NO_2-L-Trp		4-F-7-NO_2-L-Trp[c]	
	δ_C[a], type	δ_H[b] (*J* in Hz)	δ_C, type	δ_H (*J* in Hz)	δ_C, type	δ_H (*J* in Hz)
2	128.4, CH	6.88 s	-, CH		128.5, CH	7.29 s
3	109.8, C		-, C		108.3, C	
3a	124.2, C		-, C		115.2, C	
4	127.7, C		-, C		151.5, C	
5	142.2, C		-, CH	6.63 d (8.1)	119.4, CH	7.80 dd (8.1, 8.1)
6	118.7, CH	7.22 d (9.0)	-, CH	6.80 d (8.3)	108.4, CH	7.22 d (9.1)
7	109.7, CH	6.84 d (8.9)	-, C		128.6, C	
7a	138.3, C		-, C		142.3, C	
1'	171.4, C		-, C		171.3, C	
2'	53.6, CH	3.80 dd (10.1, 5.1)	-, CH	3.92 dd (10.7, 5.4)	53.7, CH	4.26 m
3'	27.7, CH_2	3.22 dd (15.6, 5.1)<br>2.84 dd (15.7, 10.1)	-, CH_2	3.22 dd (16.2, 5.4)<br>2.98 dd (16.2, 10.8)	26.7, CH_2	3.43 dd (15.2, 6.5)<br>3.32 dd (15.2, 8.2)
4-Me	15.15, CH_3	2.23 s	-, CH_3	2.10 s	-	-

[a]Data recorded at 151 MHz; [b]data recorded at 600 MHz; [c]data were from reference 23.

Table S3: Primers for gene expression

Name	Sequence (5'→3')	Function
SELKnco-F	ATACCATGGTGACCGTCCCCTCGCCG	TxtE cloning
SELKsac-R	ATAGAGCTCGCGGAGGCTGAGCGGCAG	TxtE cloning
BM3R-F	CTACATATGTCTGCTAAAAAAGTACGCAA	BM3R fusion
BM3R-R	ATCCTCGAGCCCAGCCCACACGTCTTTTG	BM3R fusion
BM3LKsac3-F	tctGAGCTCAACGCTCATAATACGCCGCTG	TxtE-BM3R fusion F primer
BM3LKsac6-F	tctGAGCTCAAGGCAGAAAACGCTCATAATACG	TxtE-BM3R fusion F primer
BM3LKsac9-F	tctGAGCTCGTACGCAAAAAGGCAGAAAACG	TxtE-BM3R fusion F primer
BM3LKsac11-F	tctGAGCTCAAAAAAGTACGCAAAAAGGCAG	TxtE-BM3R fusion F primer
BM3LKsac12-F	tctGAGCTCGCTAAAAAAGTACGCAAAAAGGCAG	TxtE-BM3R fusion F primer
BM3LKsac13-F	tctGAGCTCTCTGCTAAAAAAGTACGCAAAAAGGCAG	TxtE-BM3R fusion F primer
BM3LKsac14-F	tctGAGCTCCAGTCTGCTAAAAAAGTACGCAAAAAG	TxtE-BM3R fusion F primer
BM3LKsac15-F	tctGAGCTCGAACAGTCTGCTAAAAAAGTAC	TxtE-BM3R fusion F primer
BM3LKsac16-F	tctGAGCTCACTGAACAGTCTGCTAAAAAAG	TxtE-BM3R fusion F primer
BM3LKsac17-F	tctGAGCTCAGCACTGAACAGTCTGCTAAAAAAG	TxtE-BM3R fusion F primer
BM3LKsac19-F	tctGAGCTCTCACCTAGCACTGAACAGTCTGC	TxtE-BM3R fusion F primer
BM3LKsac22-F	tctGAGCTCGGTATTCCTTCACCTAGCACTGAAC	TxtE-BM3R fusion F primer
BM3LKsac24-F	tctGAGCTCCTTGGCGGTATTCCTTCACCTAG	TxtE-BM3R fusion F primer
BM3LKsac27-F	tctGAGCTCAAAATTCCGCTTGGCGGTATTC	TxtE-BM3R fusion F primer
BM3LKxho-R	atcCTCGAGCCCAGCCCACACGTCTTTTGC	TxtE-BM3R fusion R primer
TxtESF	CACCCATGGTGACCGTCCCCTCGCCGCTC	TxtES fusion F primer
TxtESR	CGGGTTGCGGGCGAACGC	TxtES fusion R primer
V1-3-F	GCGTTCGCCCGCAACCCGCATGTATTACAAAAAGCAGCAGAAGAAGC	JKLoop insert F primer
V1-3-R	GGCCGCGACGCGCCAAGGAGCAGTTGGCCATAAGCG	JKLoop insert R primer
FW-V1	ACCTGGCGCGTCGCGGC	S13BM3R fusion F primer
RV	GACCCAGCCCACACGTCTTTTGC	S13BM3R fusion R primer

A.

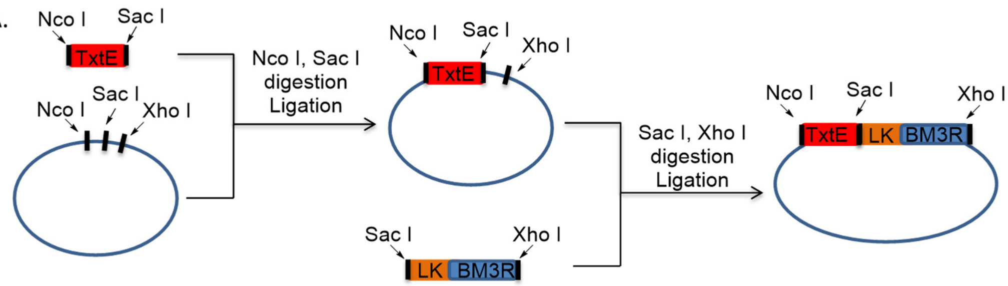

B.

Variant name	Linker length	Linker sequence
TB27	27	EL KIPLGGIPSPSTEQSAKKVRKKAEN
TB24	24	EL LGGIPSPSTEQSAKKVRKKAEN
TB22	22	EL GIPSPSTEQSAKKVRKKAEN
TB19	19	EL SPSTEQSAKKVRKKAEN
TB17	17	EL STEQSAKKVRKKAEN
TB16	16	EL TEQSAKKVRKKAEN
TB15	15	EL EQSAKKVRKKAEN
TB14	14	EL QSAKKVRKKAEN
TB13	13	EL SAKKVRKKAEN
TB13-Q	13	EQ SAKKVRKKAEN
TB12	12	EL AKKVRKKAEN
TB11	11	EL KKVRKKAEN
TB9	9	EL VRKKAEN
TB6	6	EL KAEN
TB3	3	EL N

Fig. S1 A: Schematic depiction of stepwise construction of TxtE chimeras with variable lengths. The expression vector backbone is pET28b. **B:** Detailed description of TxtE chimeras with variable linker lengths.

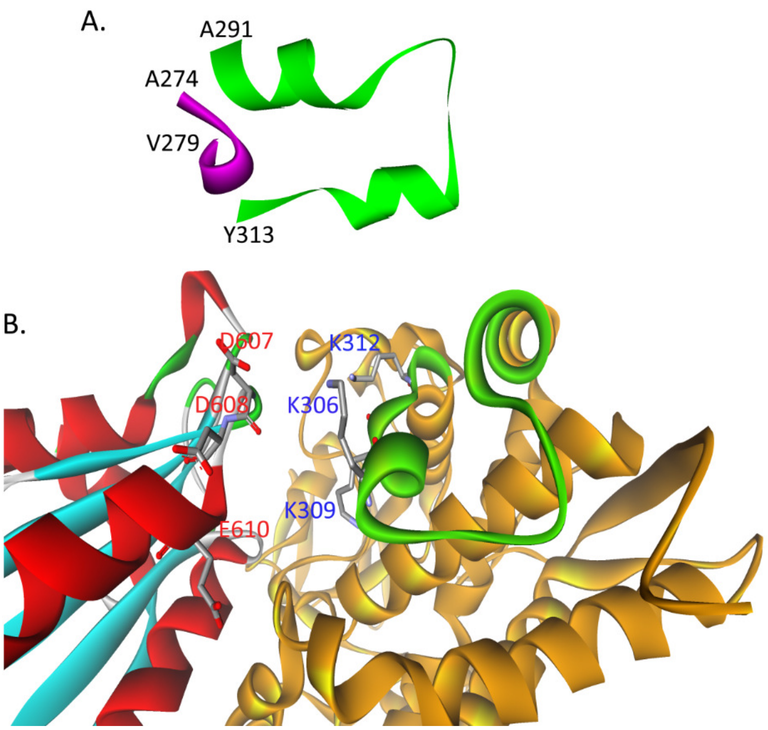

Fig. S2 A: A notable length difference at the loop connecting J and K helices was identified after superimposing crystal structures of TxtE (PDB: 4TPO) in purple and P450BM3 heme domain (PDB: 1ZO9) in green. **B:** The interface between BM3 heme and FMN-binding domains. The loop connecting J and K helices is labeled in green and its basic residues are shown as sticks. Acidic residues in the loop motif of FMN-binding domain are also shown.

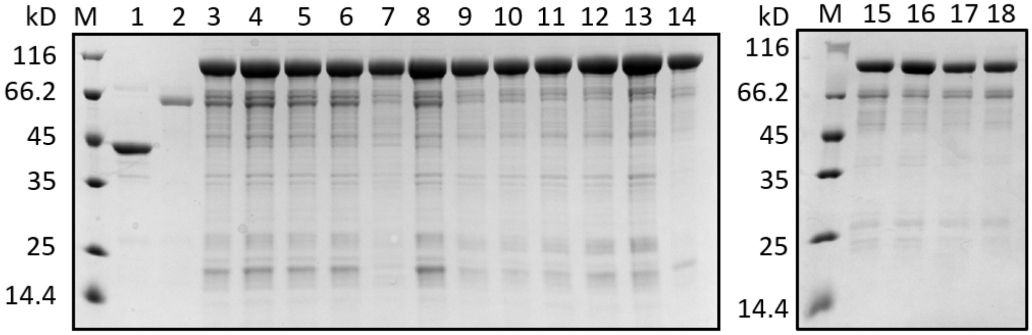

Fig. S3: SDS-PAGE analysis of purified recombinant proteins. M: protein marker; Lane 1-14: TxtE, BM3R, TB3, TB6, TB9, TB11, TB13-Q, TB14, TB17, TB19, TB22, TB24, TB27, and TB13S. Lane 15-18: TB12, TB13, TB15, and TB16.

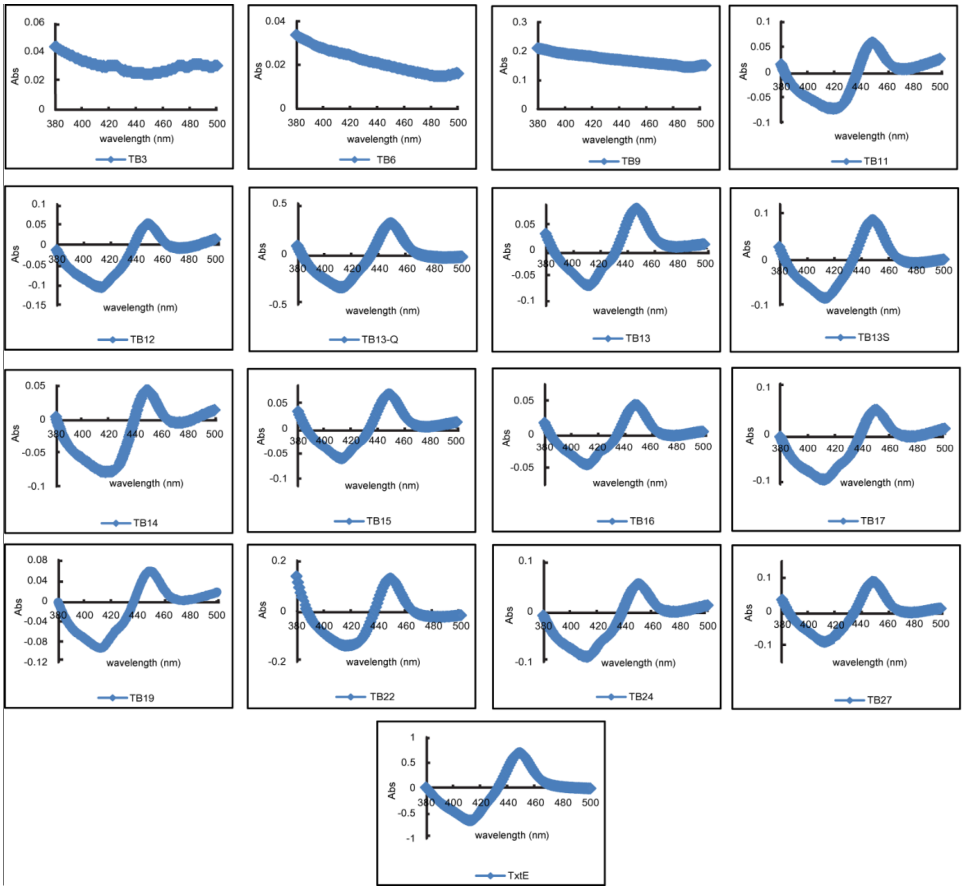

Fig. S4: CO-reduced difference spectra of chimeric TxtE fusion constructs and TxtE. A peak at around 450 nm indicates the properly folded, active P450.

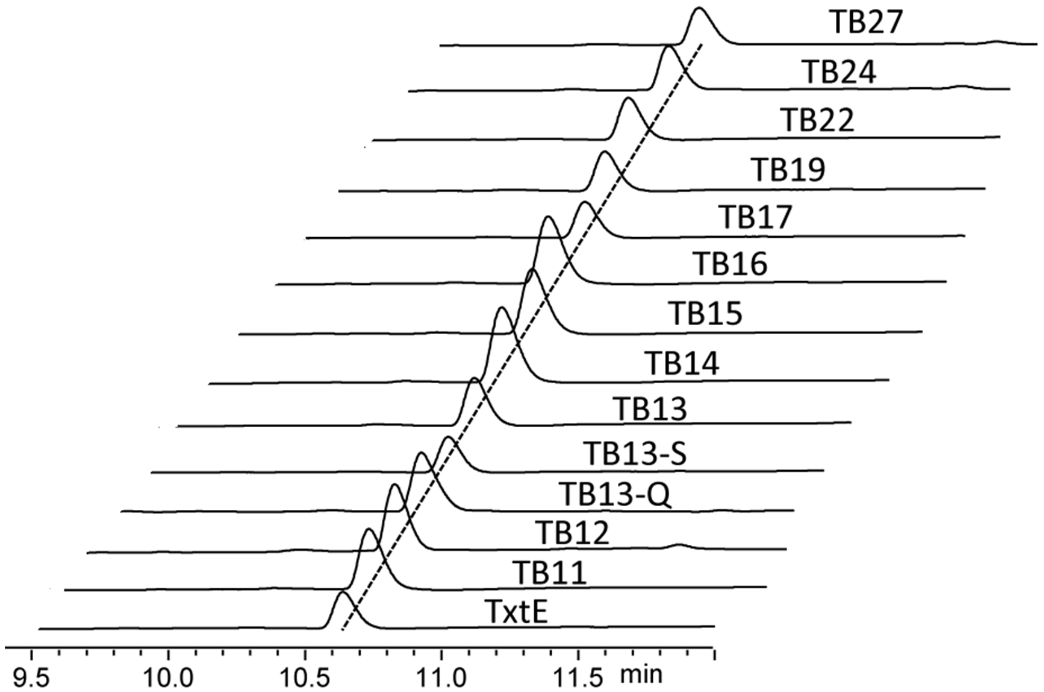

Fig. S5: HPLC spectrum of TxtE and chimeric TxtE-BM3R variants relative nitration activity assay. All reactions contained 0.5 mM Trp and 1.5 µM P450. The TxtE reaction was further supplemented with 0.43 µM spinach Fer and 0.33 µM Frd. The reactions were incubated at 20 °C, 300 rpm for 30 minutes. All experiments were repeated at least three times.

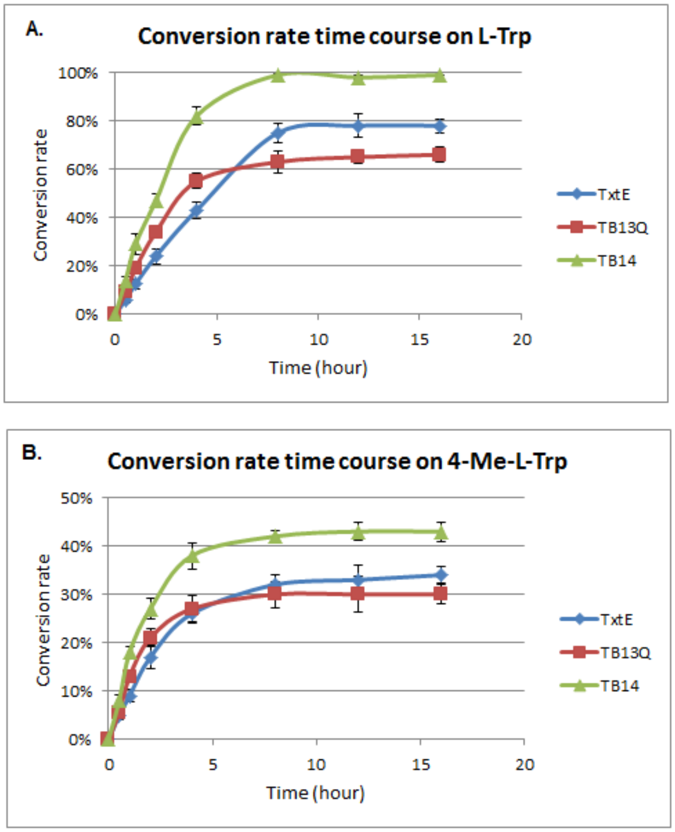

Fig. S6: Time course of TxtE, TB13Q and TB14 nitration activity assay. All reactions contained 0.5 mM Trp and 1.5 µM P450. The TxtE reaction was further supplemented with 0.43 µM spinach Fer and 0.33 µM Frd. The reactions were incubated at 20 °C, 300 rpm. All experiments were repeated at least three times.

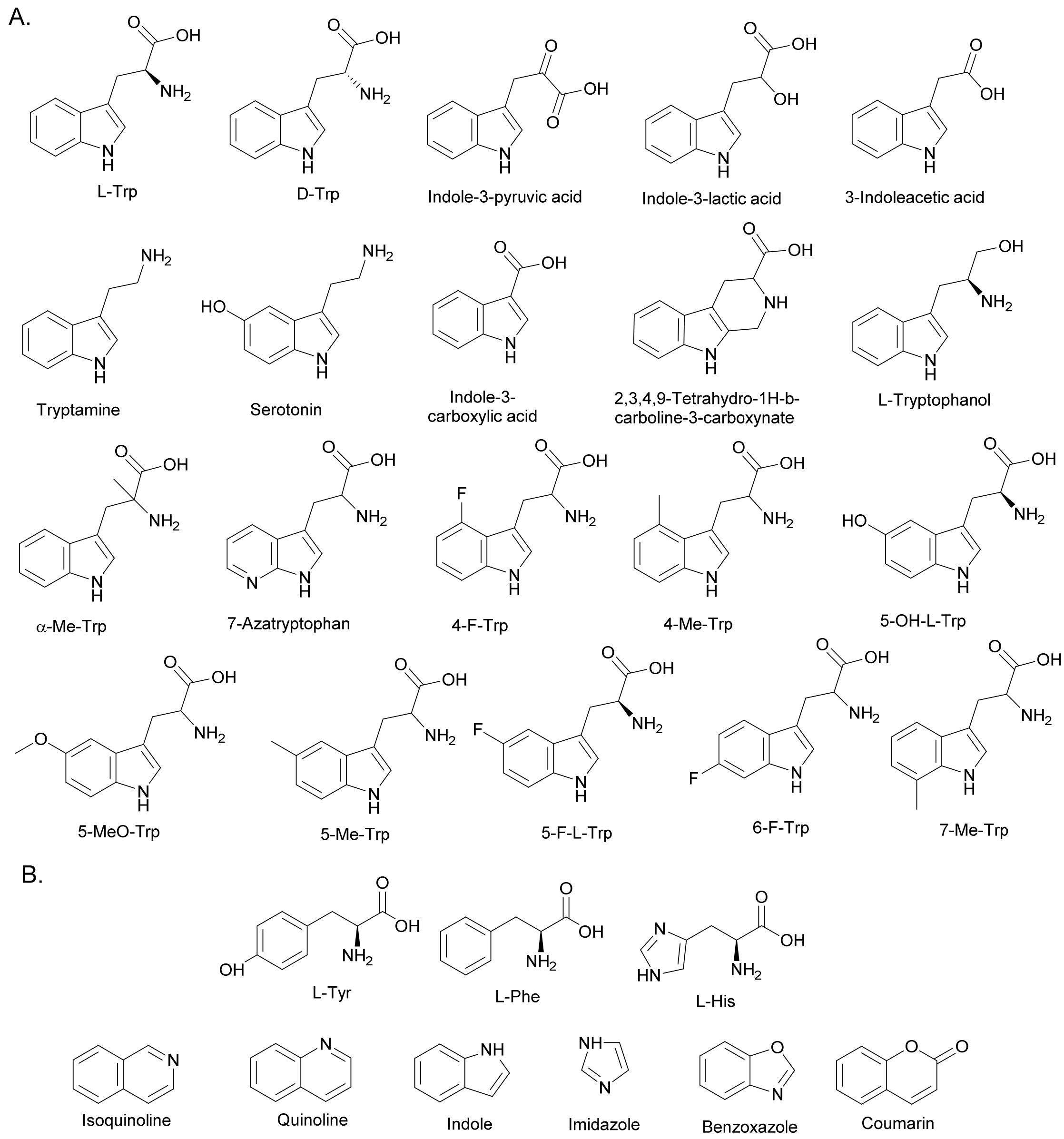

Fig. S7 A: Chemical structures of Trp and its analogs that induced type I spectral shift of TxtE and TB14. **B:** Structures of compounds that had no detectable level of interactions with TxtE and TB14.

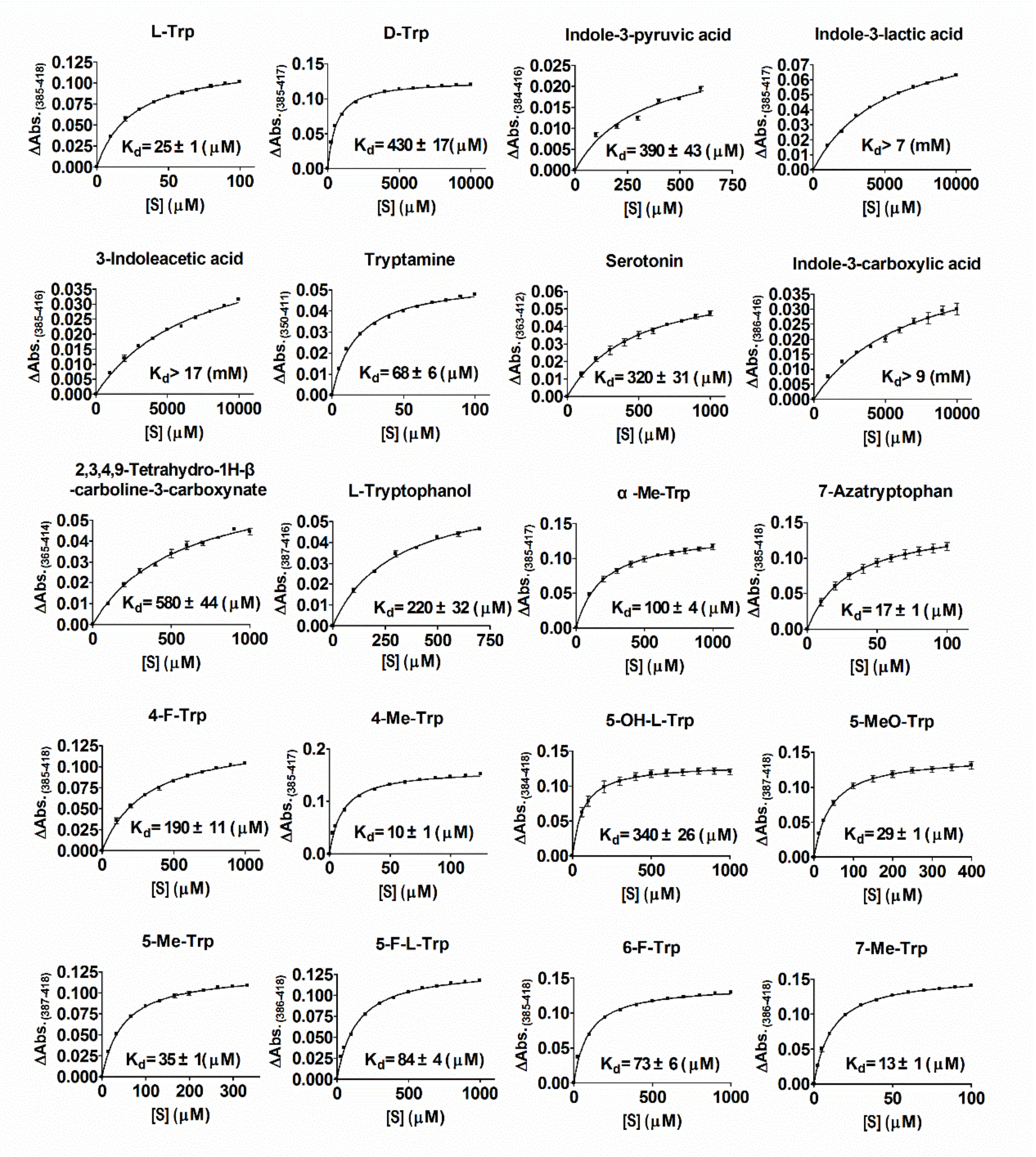

Fig. S8: Substrate binding to TxtE. All data represented means ± S.D. of three independent experiments. Data were fitted to the Michaelis-Menten equation in Prism 5.0.

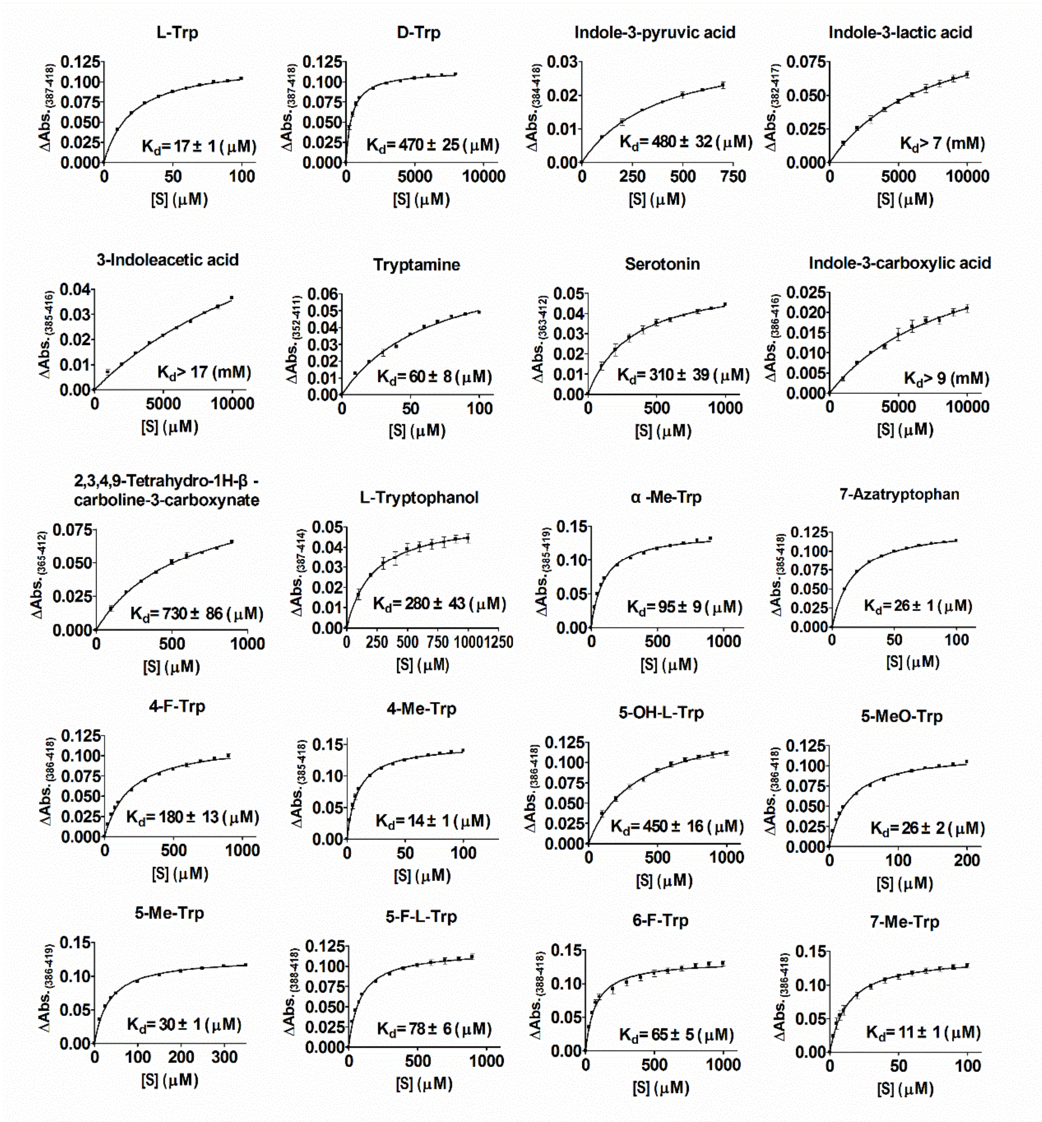

Fig. S9: Substrate binding to TB14. All data represented means ± S.D. of three independent experiments. Data were fitted to the Michaelis-Menten equation in Prism 5.0.

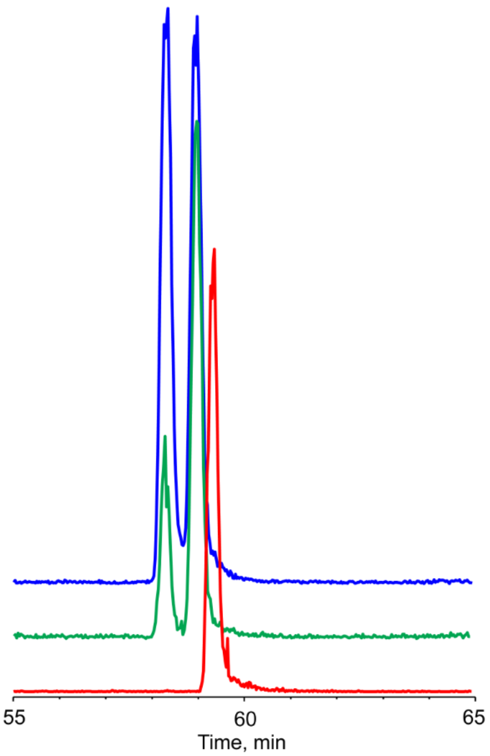

Fig. S10: LC-MS analysis of Marfey's derivatization of 4-Me-DL-Trp and its nitro product. Blue: ion extract spectrum of Marfey's derivatized 4-Me-DL-Trp; Green: ion extract spectrum of Marfey's derivatized 4-Me-DL-Trp after the enzyme reaction; and Red: ion extract spectrum of Marfey's derivatized nitro product.

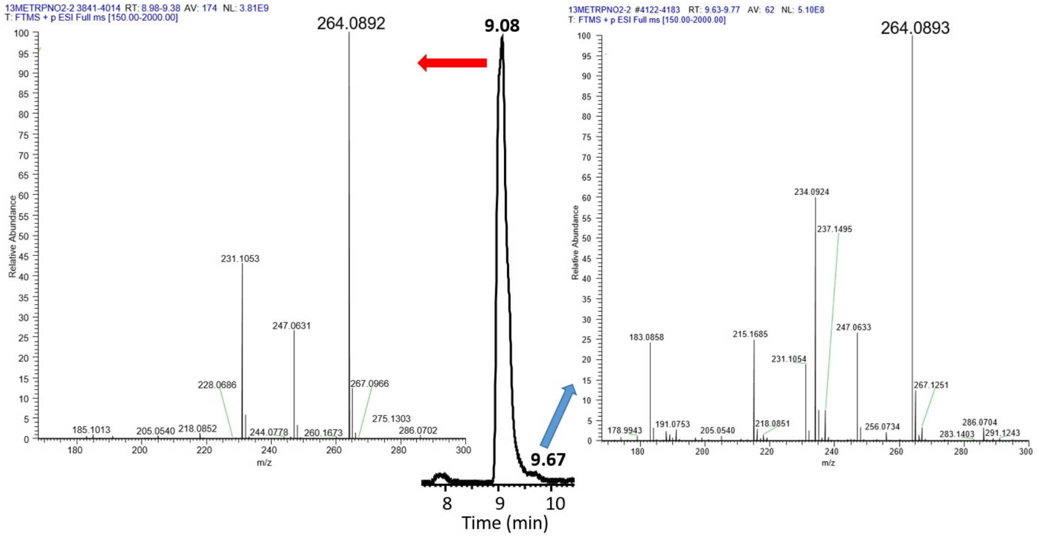

Fig. S11: LC-HRMS analysis of the isolated nitro product. Two peaks with the retention times of 9.08 min and 9.67 min showed the same m/z value of about 264.0892.

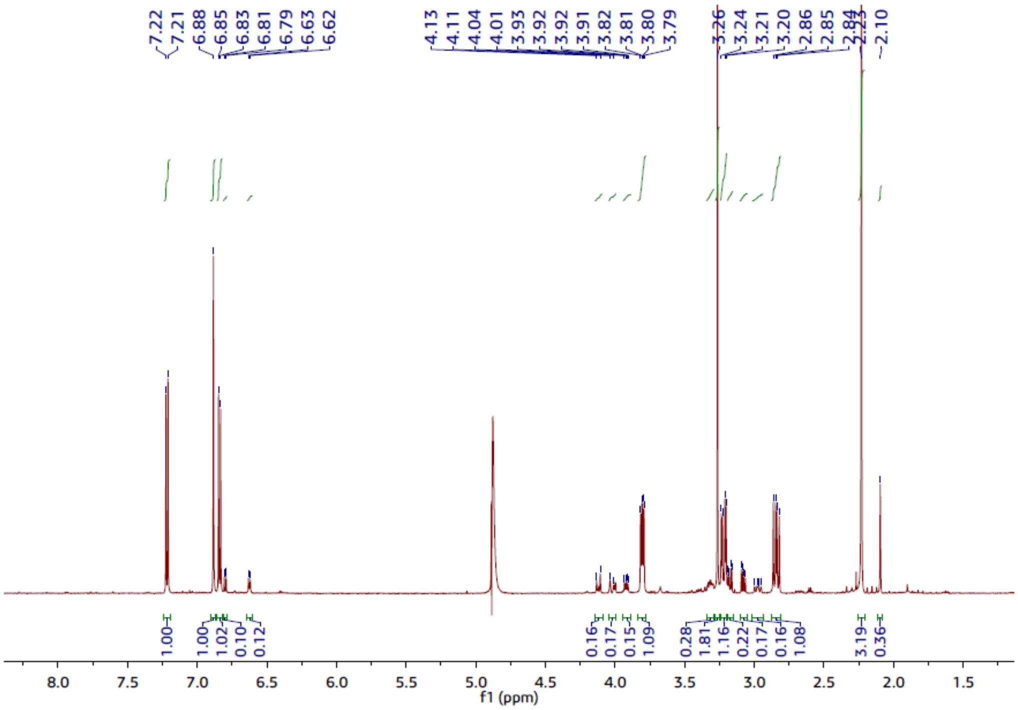

Fig. S12: ^{1}H NMR spectrum of the isolated nitro product.

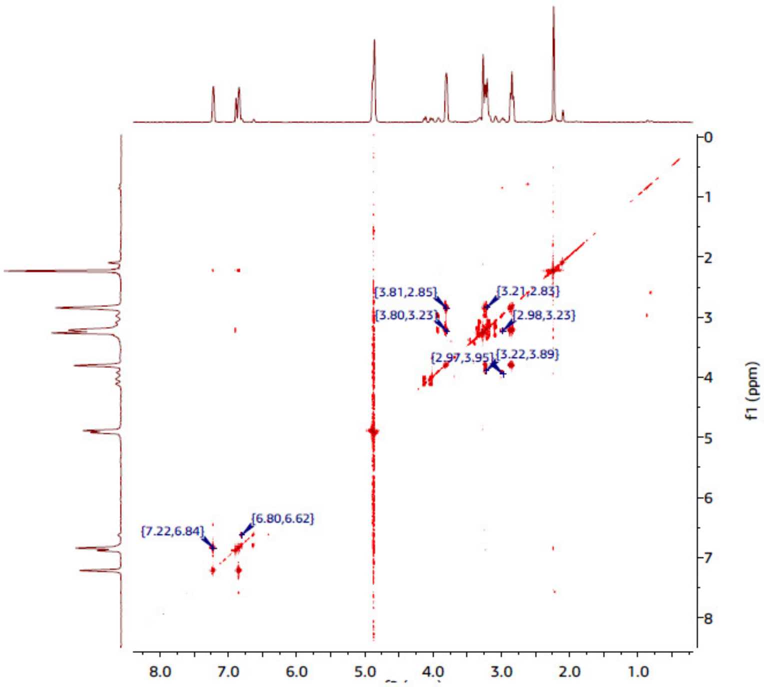

Fig. S13: COSY spectrum of the isolated nitro product.

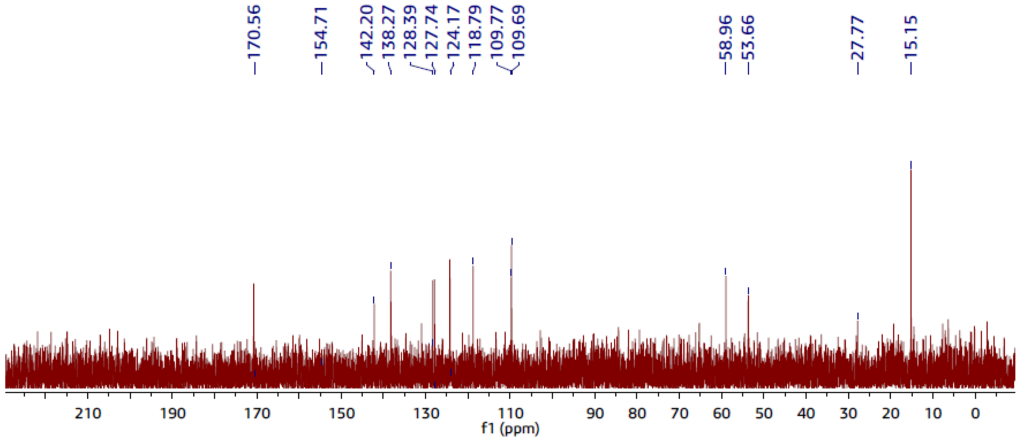

Fig. S14: ^{13}C NMR spectrum of the isolated nitro product.

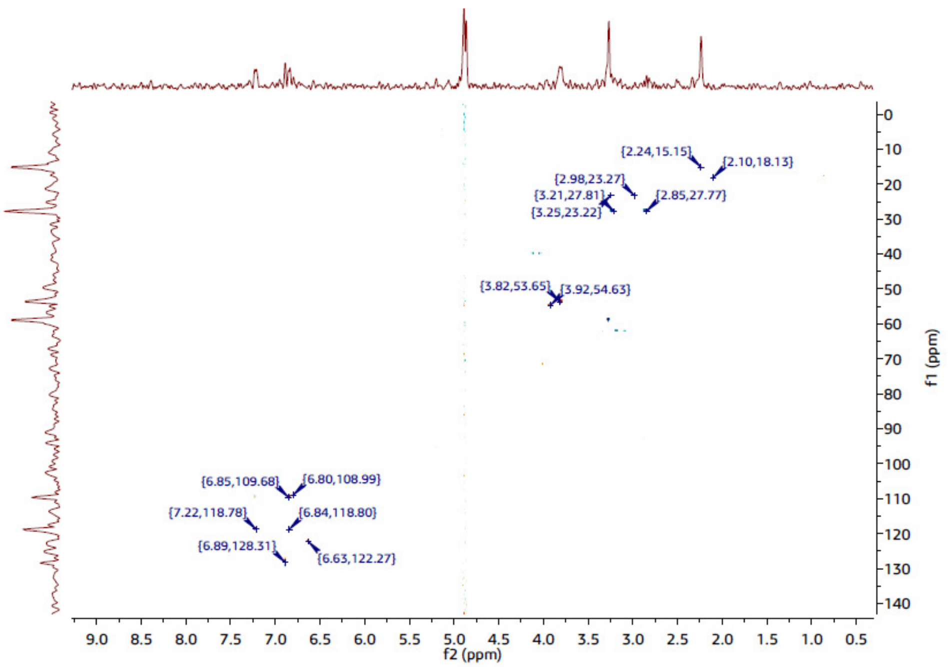

Fig. S15: HSQC spectrum of the isolated nitro product.

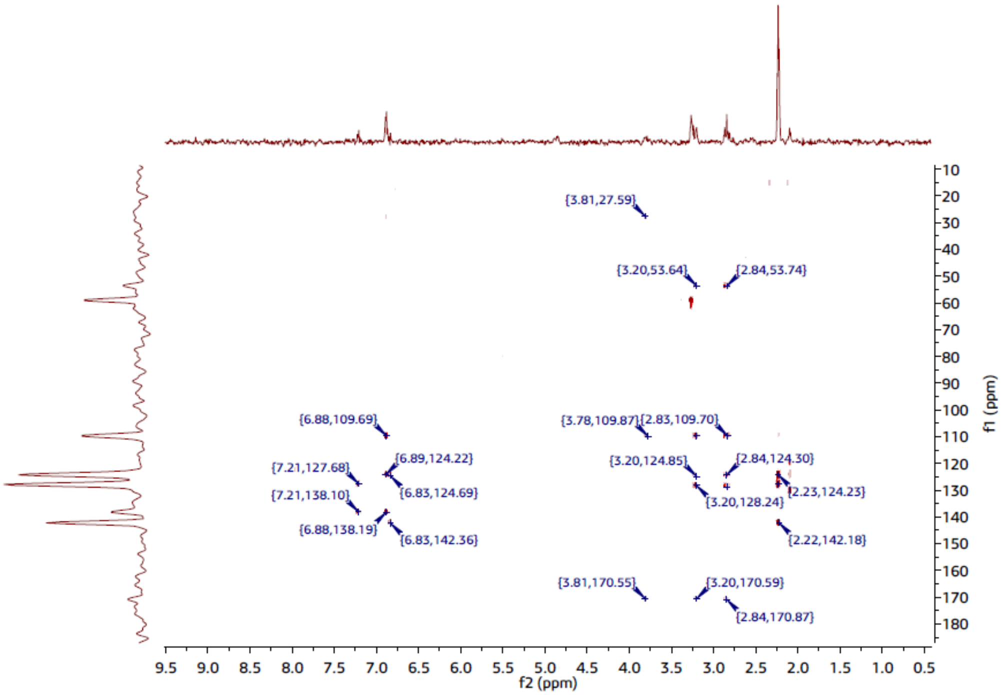

Fig. S16: HMBC spectrum of the isolated nitro product.

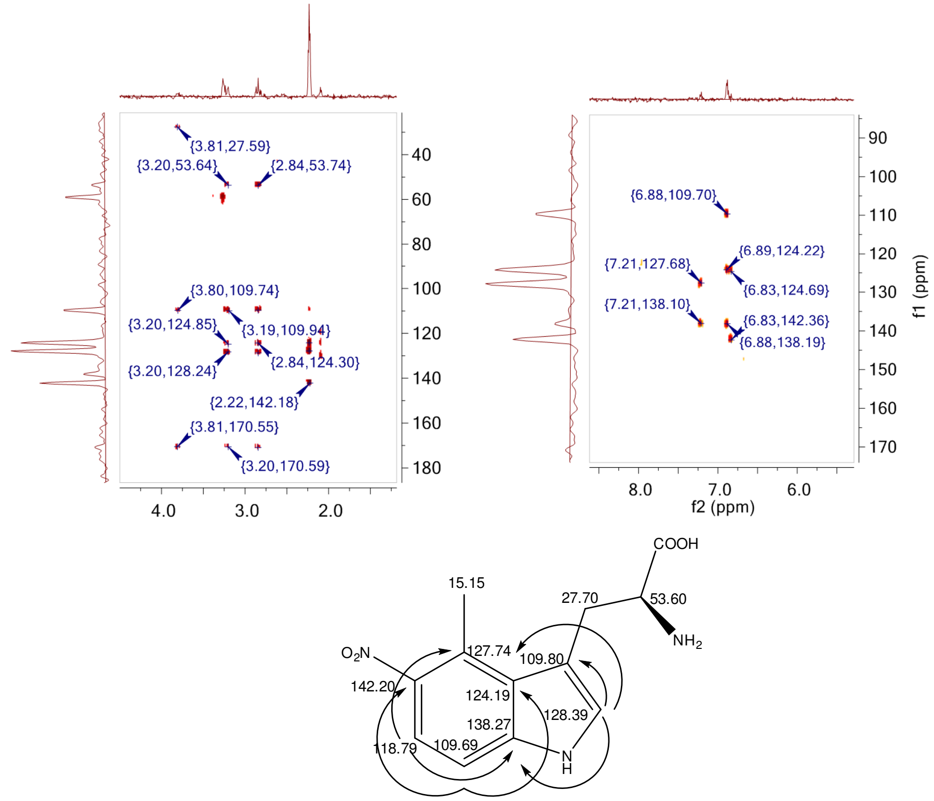

Fig. S17: Expansion of HMBC spectrum in aromatic and aliphatic regions and the representative correlations on 4-methyl-5-nitro-L-tryptophan.
